# Alleviating head-mounted weight burden for neural imaging in freely-behaving rodents

**DOI:** 10.1038/s41598-025-04300-0

**Published:** 2025-05-31

**Authors:** Yuehan Liu, Jing Zhang, Cheng-Yu Lee, Haolin Zhang, Xingde Li

**Affiliations:** 1 Department of Electrical and Computer Engineering, Johns Hopkins University, Baltimore, MD 21218 USA; 2 Department of Biomedical Engineering, Johns Hopkins University, Baltimore, MD 21205 USA

**Keywords:** Biomedical engineering, Biophotonics

## Abstract

The recently developed miniaturized head-mounted two-photon (2P) imaging devices have served as a valuable tool for neuroscientists, enabling real-time functional neural imaging in freely-behaving animals. Although the current 2P fiberscopes and miniscopes are lightweight, the weight of any potential additional accessories inevitably imposes a burden on the animal. Here, we present a buoyancy levitation method to alleviate head-mounted weight burden on mice. By utilizing the buoyance of a helium-filled balloon to counteract the additional weight of up to 7 g, mice’ motion behavior remains largely unaffected by the added load. Neuroimage analysis provides new insights into the effects of weight burden on neural activities. This easy-to-implement method offers a platform for studying neural network function in animals, effectively freeing them from the burden of head-mounted weight.

**Supplementary Information:**

The online version contains supplementary material available at 10.1038/s41598-025-04300-0.

## Introduction

Two-photon (2P) fluorescence microscopy has served as a valuable tool in neuroscience for investigating dynamic neural networks with high spatiotemporal resolution^1–3^. Along with genetically encoded calcium indicators, particularly the family of GCaMP, benchtop 2P microscopy has been widely used to optically assess neural activities in head-fixed animals^4–7^. While effective, these protocols are suboptimal for studying nonstationary behaviors such as grooming, spatial navigation, and social interaction. Additionally, head fixation induces pressure and fatigue in the head and neck, which can lead to aberrant neural feedback, and interfere with the study of neural circuit function.

To address these limitations, miniaturized, head-mountable 2P imaging devices have been developed, allowing the visualization of brain activities and neural network dynamics in freely-behaving animals^8–10^. Over the past two decades, significant efforts have been invested to reduce the size and weight of 2P miniscopes^11–13^. Recently developed ultralight ultracompact 2P fiberscopes (weighing 1 g, with an outer diameter of 2.4–2.8 mm) have enabled neuroimaging in freely-walking/rotating mice^14–18^. However, despite the lightweight design of current 2P fiberscopes and miniscopes, additional optics or mechanical components may need to be attached to the imaging probe for specific imaging control purposes, such as light-targeting devices for optogenetics control^19,20^. This added weight of the accessories inevitably places a burden on the animal’s head, potentially affecting its behavior.

In this study, we developed and demonstrated a buoyancy levitation method to alleviate the head-mounted weight burden in freely-behaving mice. By introducing a buoyant force to the head-mounted fiberscope using a standard helium balloon (15 L), we demonstrated the weight cancellation of the additional accessories on the mouse’s head, covering a range of 0–7 g. Motion behavioral analyses revealed that the mice exhibited similar freedom of walking (in terms of walking distance, turning angle, linear/angular velocity and acceleration) when the balloon was used to lift the head-mounted weight, compared to when only the bare fiberscope was worn by the mice. Furthermore, investigation of the animal’s neural activities indicated that the weight burden interfered with the firing activities of some neurons. We believe this convenient method offers a practical solution to reduce the head-mounted weight on animals and enable neural network function study when the animal is under naturalistic conditions.

## Methods

### Buoyancy levitation system

**Fig. 1:**
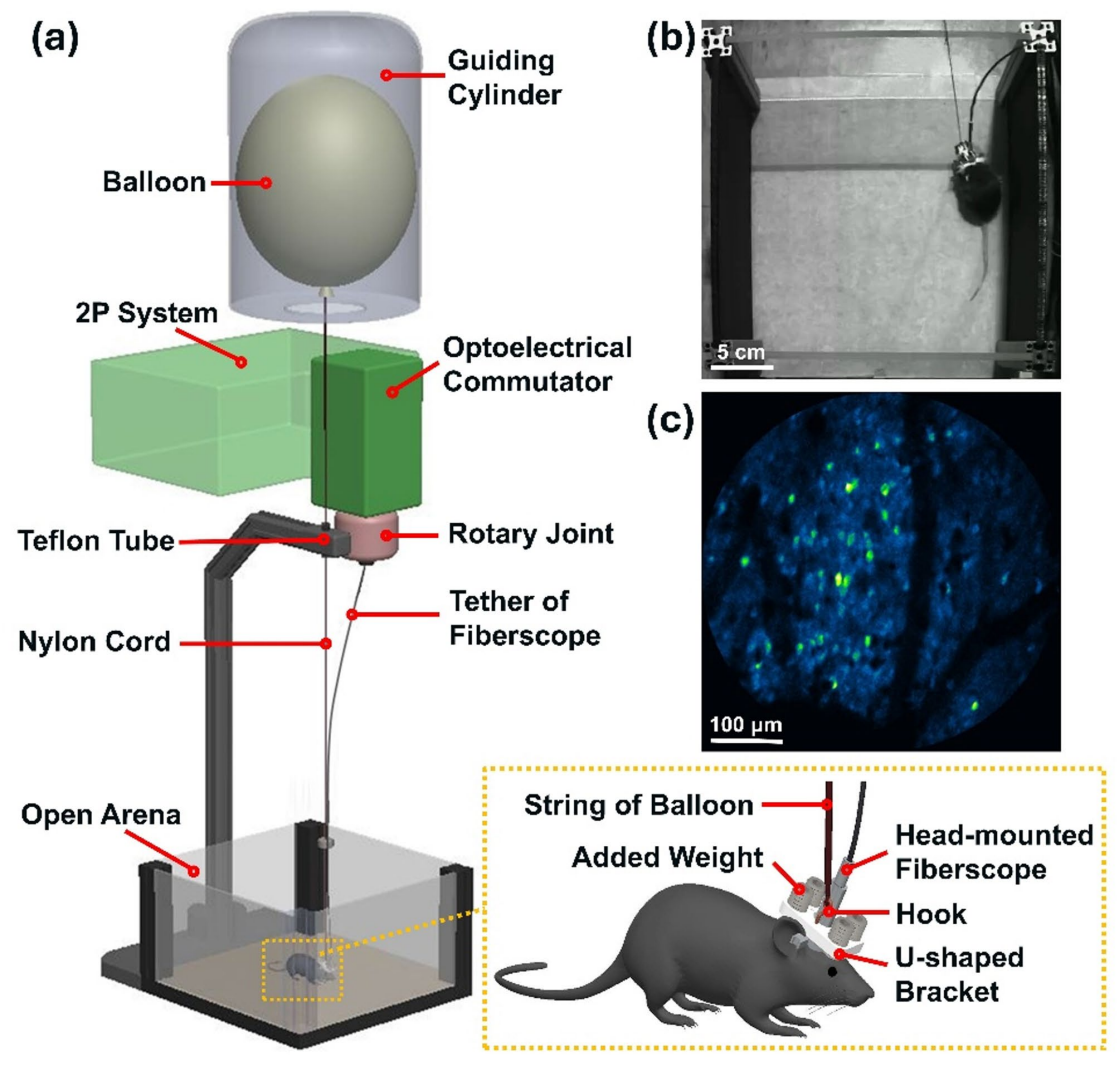
The buoyancy levitation system for 2P neural imaging in freely-behaving mice. (**a**) Schematic of the buoyancy levitation system. Lower-right: A zoomed-in diagram of the mouse’s head with a 2P fiberscope and an additional weight. (**b**) Photograph of a mouse in an open arena with a head-mounted fiberscope, additional weight, and a balloon counteracting the weight. (**c**) Representative 2P image of neurons expressing GCaMP6m, obtained from a fiberscope.

To reduce the head-mounted weight burden imposed on the freely-behaving mice, we developed a buoyancy levitation system for 2P neural imaging. The schematic of the system is shown in Fig. 1a, where a helium balloon, filled with an 80% helium–20% air mixture, provides lifting buoyancy to counteract the additional weight placed on the mouse’s head. A guiding cylinder is positioned above to restrict the lateral movement of the balloon, thereby minimizing the air resistance when the animal moves and the associated vertical movement of the balloon. A piece of Teflon tubing is installed at the connection point between the nylon cord and the stand to reduce friction and further ensure the vertical-only movement of the balloon. The block diagram represents our 2P imaging platform, where the 2P fiberscope is connected to an electrical commutator for rotational tracking and compensation, allowing neuroimaging in freely-walking/rotating mice^16^. Our fully integrated 2P fiberscope (with an outer diameter of 2.8 mm and weighing 1.3 g) is based on fiber-optic scanning technology^17,21–27^ and our recent composite fiber cantilever design for obtaining a large field of view (FOV)^14^. Details of the 2P system and fiberscope are provided in Supplementary Materials (Fig. S1). Zoomed-in diagram of the mouse’s head is shown in the lower right, where a 3D-printed lightweight U-shaped bracket (0.5 g) is designed for easy mounting of additional weight (e.g., stainless metal rods). The balloon could hook onto this U-shaped bracket to provide buoyancy without interfering the attachment of the fiberscope. Figure 1b shows a mouse in the arena with a head-mounted fiberscope and a balloon counteracting the additional weight. Figure 1c provides a representative 2P GCaMP6m neuroimage acquired with the fiberscope from a freely-behaving mouse.

The buoyancy of the helium balloon $F$ can be calculated using the following equation:

$$F= \Delta \rho \cdot g\cdot V=\left({\rho }_{air}-{\rho }_{balloon}\right)\cdot g\cdot V,$$

where ${\rho }_{air}$ is the density of air, ${\rho }_{balloon}$ is the density of the gas inside the balloon (80% helium–20% air mixture), $g$ is the gravitational acceleration, and $V$ is the volume of the balloon.

A standard party balloon can be inflated into an approximately 15 L ellipsoid. According to our calculation, the buoyancy it provides can lift around 12 g of weight. The balloon itself weighs 3 g, and with the addition of a nylon cord and a hook, the total weight is approximately 5 g. Therefore, the buoyancy levitation system with a 15 L balloon is capable of offsetting an additional weight of up to 7 g. A helium balloon of large size could be used to lift a heavier weight. We measured the relationship between the weight cancelled by the buoyant force and the volume of the balloon. The actual measurement results closely match the theoretical predictions. See Fig. S2 in Supplementary Materials for details.

### Animal model

Three Camk2-Cre line mice (Jax, #005359) with GCaMP6m expressed in the somatosensory cortex were used in this work. All experimental studies were in accordance with ARRIVE guidelines. All experimental methods were approved by the Johns Hopkins University Animal Care and Use Committee under animal protocol #MO21M4111. All methods were performed in accordance with the relevant guidelines and regulations.

### Cranial window surgery

At the age of 8-weeks old, the mice were anesthetized by inhalation of 2% isoflurane and O_2_ and then locked to a stereotaxic platform. After removing the scalp and exposing the skull, a 4 mm diameter round craniotomy was drilled over the somatosensory cortex of the mice. AAV/DJ-flex-GCaMP6m virus (Neuroconnectivity Core, Baylor College of Medicine) was injected into the target region (− 2.5 mm lateral, − 1.2 mm anterior to the bregma, 0.55 mm depth) via a glass microneedle (World Precision Instrument TIP10LT, 1 mm O.D., 10 µm tip diameter) attached to a microinjector pump (Nanoject II, Drummond). After the injection, a 100 µm thickness glass coverslip was placed to the exposed brain, and it was sealed to the skull with tissue adhesive (3 M Vetbond). A customized titanium head-restraining bar was glued to the head with dental cement for later attaching the endomicroscope. The transgene expression was checked 3–4 weeks post surgeries with a tabletop 2P microscope. Enthanasia (CO_2_) would be performed only if the cranial window showed signs of infection (e.g., cloudy window) or the condition or health of the mouse degraded beyond recovery. Every effort was made to reduce the number of animals used, and to minimize their suffering.

### Neural imaging with 2P fiberscope in freely-behaving mouse

After the transgene expression was confirmed, the mice would be used for 2P fiberscopy imaging in vivo. To begin the imaging procedure, the mouse was lightly anesthetized (0.5–2% Isoflurane + 1.5 L/min oxygen) and restrained by locking its head-restraining bar to a home-made holding platform. Then a 2P fiberscope was mounted on a 3D translational stage and carefully positioned above the cranial window to image the brain. After a suitable FOV was identified, the fiberscope was fixed onto the head-restraining bar via a customized light-weight mini-adapter, the mouse was then released from the holding platform and started moving freely in a home-built imaging platform (10″ $\times$ 10″ open arena). The mouse was allowed sufficient time to recover from anesthesia before freely-behaving neural imaging experiments. One camera (CM3-U3-13S2M-CS, FLIR) was set above the imaging platform to obtain the top view of the freely-behaving mouse under IR light illumination. The 2P imaging data from the fiberscope and behavior recordings from the camera were collected in synchronization and saved for later analysis. After imaging, the mouse will be anesthetized again, and the fiberscope was detached from the mouse. Afterwards, the mouse will be placed in a housing facility for at least 24 h of rest before the next imaging experiment.

Note that pre-experimental acclimation training was performed on each mouse to initiate the experiment. The animals were trained to become accustomed to being locked by its head-restraining bar, wearing a fiberscope and familiarized with the arena prior to the experiments, with acclimation training conducted once daily for three consecutive days.

### Neural data processing

The GCaMP fluorescence neural data was processed using MATLAB and Python. We first applied the algorithm NoRMCorre^28^ to correct motion artifacts in all images acquired by the 2P fiberscope. Since the FOV of the 2P neural images from different sessions along different days might be slightly different, we employed a non-rigid registration method TPAT^29^ to register multi-session imaging stacks using common landmarks (paired image features like blood vessels, hollow areas, etc.). Then we used a well-established pipeline CaImAn^30^ to segment neurons and extract their temporal calcium dynamics (ΔF/F traces). Finally, we analyzed neural activities based on our customized Python codes. In our analysis, the fluorescence rate of a neuronal firing was defined as the area under a firing peak within the ΔF/F trace divided by firing duration time, i.e., $\frac{\Delta S}{\Delta t}$. And the overall fluorescence rate of a neuron was calculated as the average of all firing peaks’ fluorescence rates, given by $\left(\sum_{i}^{n}\frac{\Delta {S}_{i}}{\Delta {t}_{i}}\right)/n$.

## Results

### Buoyance levitation system performance

To test the performance of the buoyancy levitation system, we studied the motion behavior of five freely-behaving mice under three conditions: (A) Probe-only: Only a 2P fiberscope was mounted on the mouse’s head. (B) Balloon + Weight: In addition to a fiberscope, an additional 6 g additional weight was mounted on the mouse’s head, with a helium balloon providing enough buoyancy to cancel out the added weight. (C) Weight: A fiberscope and a 6 g additional weight were mounted on the mouse’s head, but without any buoyant lift. Here, we chose a weight of 6 g (~ 25% of a mouse’s body-weight) to test the effectiveness of the buoyancy system in reducing the extremely heavy weight burden on mouse’s head. Note that mice without wearing any probes can be an ideal reference case. However, considering that factors beyond weight—such as the size, position, and tethers of head-mounted devices—can also influence mouse behavior, we believe that mice without any devices are not a suitable comparison for this experiment. Therefore, we used the 'probe-only’ condition as the control instead of mice wearing nothing.

During the experiment, each mouse was set free in an open arena for a session of 167 s (corresponding to 500 frames for a 2P fiberscope with an imaging speed of 3 frames/s) under each of the three conditions. Between sessions, there was an intersession break of at least 5 min to allow the animal sufficient rest. The animal’s motion behavior was recorded using a camera and the walking trajectories were extracted from the videos using DeepLabCut^31^, a toolbox for motion tracking. From these trajectories, the total walking distance and turning angle of the animal were calculated using customized codes in Python.

**Fig. 2:**
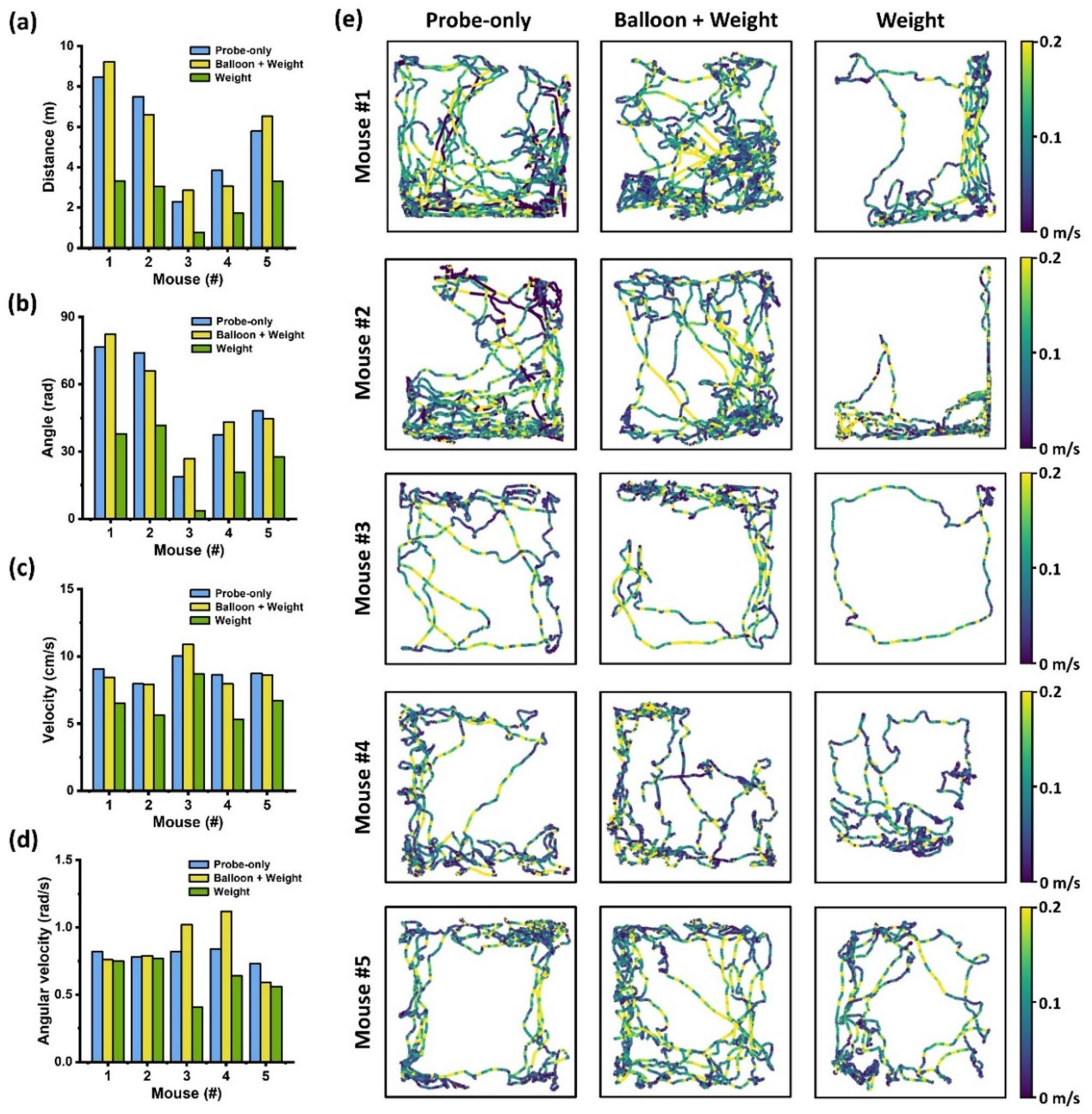
(**a**) Total walking distance within a session under different conditions (blue: probe-only, yellow: balloon + weight, green: weight) of five mice (Mouse #1–#5). (**b**) Total turning angle within a session under different conditions of the five mice. (**c**) The average velocity during walking periods under different conditions of the five mice. (**d**) The average angular velocity during walking periods under different conditions of the five mice. (**e**) Movement trajectories within each session of the five mice. Color indicates the moving velocity of the mouse. (left: probe-only, middle: balloon + weight, right: weight).

The total walking distance for all five mice during each session under the different conditions is shown in Fig. 2a, with the total turning angle presented in Fig. 2b. For all mice, the walking distances are greater under the “probe-only” and “balloon + weight” conditions compared to the “weight” condition, with mean distances of 5.57 m, 5.66 m, and 2.43 m, respectively. A similar pattern is observed for the turning angles, with mean angles of 51.00 rad, 52.58 rad, and 26.35 rad, respectively.

To investigate the effects of different conditions on mouse movement while accounting for individual differences between mice, we used a mixed-effects model for statistical analysis (conducted in SPSS® (by IBM), with a significance level set at 0.05). In the mixed-effects model, the three conditions were set as fixed effects, and the five mice were set as random effects. The analysis showed that overall, these three different conditions had significant impacts on the movement of the mice in terms of both walking distance (*p* = 0.0003) and turning angle (*p* = 0.0001), while individual differences among mice were not significant (walking distance: *p* = 0.1462, turning angle: *p* = 0.1278). Pairwise comparisons of the effects of the three conditions were then performed, with p-values from Bonferroni post hoc tests presented in Tables 1 and 2. The results indicate that there is no significant difference in the movement state (both walking distance and turning angle) of the mice between the “probe-only” and “balloon + weight” conditions. However, when comparing “probe-only” vs. “weight” or “balloon + weight” vs. “weight,” significant differences in mice’s movement were observed.

**Table 1:** Mice’s walking distance analysis: *p*-values from Mixed model (left) and Bonferroni post hoc tests (right).

Mixed model	*p*-value	Bonferroni post hoc tests	*p*-value
Fixed effect^a^	0.0003	Probe-only versus Balloon + Weight	1
Random effect^b^	0.1462	Probe-only versus Weight	0.0008
		Balloon + Weight versus Weight	0.0007

^a^Fixed effect: the three conditions (Probe-only, Balloon + Weight, and Weight).

^b^Random effect: the five mice.

**Table 2:** Mice’s turning angle analysis: *p*-values from Mixed model (left) and Bonferroni post hoc tests (right).

Mixed model	*p*-value	Bonferroni post hoc tests	*p*-value
Fixed effect^a^	0.0001	Probe-only versus Balloon + Weight	1
Random effect^b^	0.1278	Probe-only versus Weight	0.0002
		Balloon + Weight versus Weight	0.0001

^a^Fixed effect: the three conditions (Probe-only, Balloon + Weight, and Weight).

^b^Random effect: the five mice.

Moreover, time periods corresponding to locomotion or non-walking (grooming or resting) were defined by applying a velocity threshold. Within the walking period, we calculated the average walking velocity and average angular velocity of all mice, which are provided in Fig. 2c and d. Similarly, using the mixed-effects model, the analysis revealed that different conditions significantly affected the mice’s movement velocity (*p* = 2e−6), while individual differences among the mice were not significant (*p* = 0.1326). Specifically, the mouse movement speed under the “weight” condition was significantly reduced compared to the “probe-only” condition (*p* = 5e−6) and the “balloon + weight” condition (*p* = 8e−6). Additionally, although the angular velocity of all mice under the “weight” condition showed varying degrees of reduction compared to the “probe-only” and “balloon + weight” conditions, this decrease was not statistically significant.

Movement trajectories of the five mice under three conditions are illustrated in Fig. 2e, with color indicating their walking velocity. Overall, all five mice exhibited similar freedom of movement when only wearing a probe or when a balloon was used to lift the additional weight, displaying more movement within the open arena. In contrast, the mice walked less and tended to rest in a certain corner of the arena when subjected to the head-mounted weight burden.

### Potential impact of weight burden on mouse motion and neural activity

To investigate the potential impact of head-mounted weight burden on both the mouse’s motion behavior and neural activities, we performed neuroimaging on the same animal (mouse #5) for three repeated experiments. During the experiment the mouse was set free in the arena for an imaging session of 167 s (500 frames of 2P images) under each of the three conditions: “probe-only”, “balloon + weight” and “weight”. Real-time in vivo neural images over the somatosensory cortex (expressing GCaMP6m) of the freely-behaving mouse were acquired using the 2P fiberscope. Both the camera video footage of the mouse and the 2P images were collected and synchronized.

**Fig. 3:**
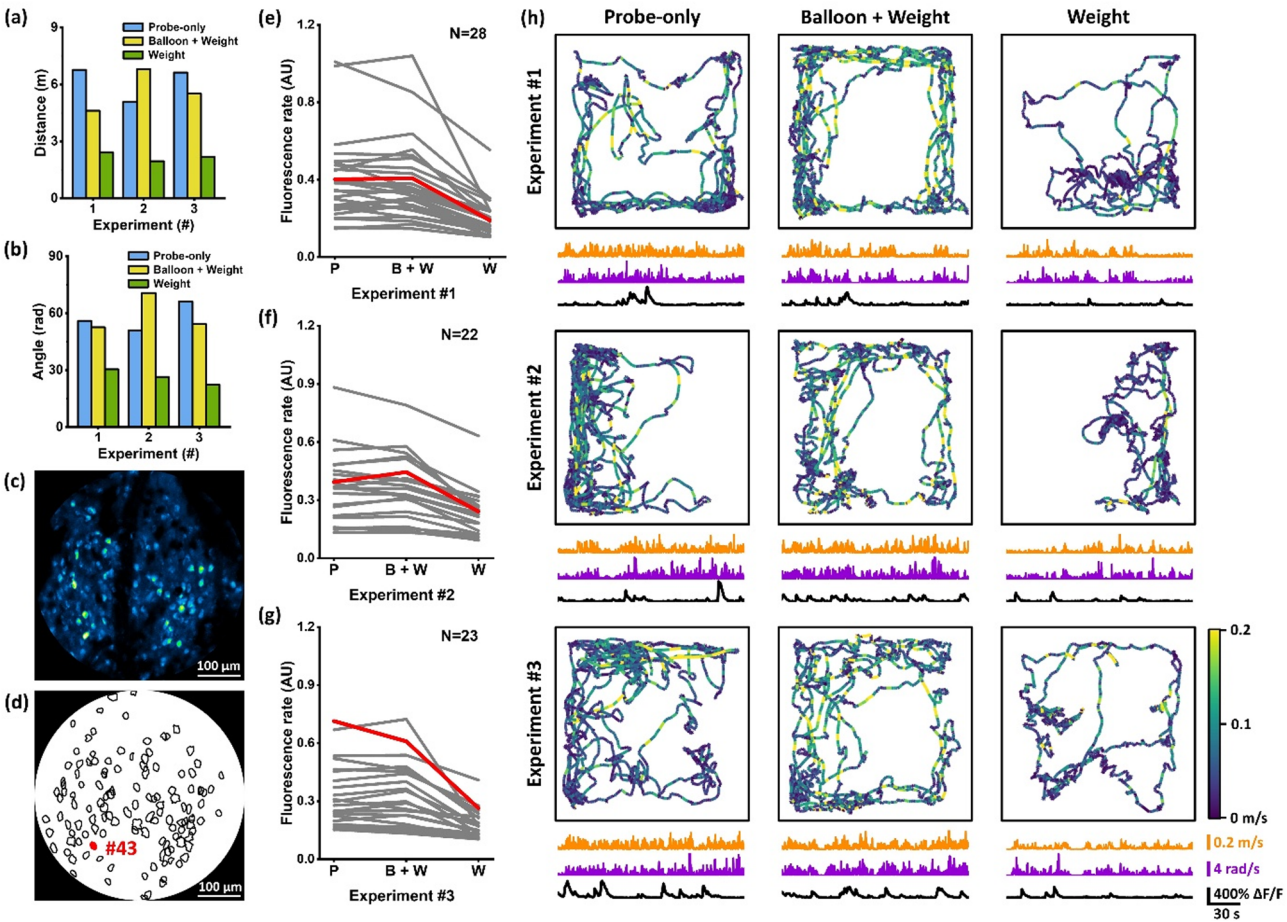
(**a**) Total walking distance within a session under different conditions (blue: probe-only, yellow: balloon + weight, green: weight) of mouse #5 during the three experiments. (**b**) Total turning angle within a session under different conditions of the mouse. (**c**) A maximal intensity projection of 500 frames of neurons (expressing GCaMP6m) in somatosensory cortex. (**d**) Segmentation mask of 124 neurons identified in the FOV after non-rigid registration. (**e**–**g**) Fluorescence rates of neurons under three different conditions (P: probe-only; B + W: balloon + weight; W: weight). These neurons did not show significant differences in their fluorescence rates under the “probe-only” and “balloon + weight” conditions, while showing obvious decrease under the “weight” condition. The numbers of these neurons in three experiments are 28, 22, and 23, respectively. The red lines are the fluorescence rates of neuron #43 whose mask is highlighted in (**d**). (**h**) Motion behavior and neural activities of the mouse within each session during three experiments. From up to down display the movement trajectories (color indicates the moving velocity of the mouse), moving velocity traces (orange), angular velocity traces (purple) and the ΔF/F traces of neuron #43 (black).

The total walking distance and turning angle of the mouse within each session are shown in Fig. 3a and b. Overall, the mouse exhibited greater movements when only wearing a fiberscope (“probe only”) or with a balloon lifting the additional weight (“balloon + weight”) than having a head-mounted weight but without buoyancy lifting (“weight”), as shown in the mean travelled distances (i.e., 6.16, 5.65 and 2.18 m, respectively, under the “probe-only”, “balloon + weight” and “weight” conditions) and the mean angle (i.e., 57.63, 59.14 and 26.40 rad, respectively, under the three conditions). Movement trajectories of each session are displayed in Fig. 3h, with the mouse’s linear and angular velocity traces plotted below. These traces revealed that the mouse was not only more active but also moved and turned faster when relieved from the 6-g head-mounted weight burden. In addition, we calculated the mouse’s linear and angular accelerations for all sessions, whose distributions are similar to the velocities (see Figs. S3 and S4 in Supplementary Materials). As indicated by acceleration, the mouse was more likely to change its movement state (accelerating or decelerating) under the “probe-only” and “balloon + weight” conditions, while its freedom of movement was reduced under the “weight” condition.

Neural activities of the mouse were investigated based on the calcium dynamic signals. Throughout the three experiments, 124 neurons were identified within a 500 µm diameter FOV. Figure 3c shows a maximal intensity projection of 500 frames of 2P neuroimages and Fig. 3d presents the segmentation masks of these neurons. The fluorescence rates of each neuron were calculated based on their ΔF/F traces. We compared the differences in each neuron’s fluorescence rate under three conditions (“probe-only”: ${r}_{P}$, “balloon + weight”: ${r}_{B+W}$, and “weight”: ${r}_{W}$). We observed some neurons whose fluorescence rates did not show significant difference between the “probe-only” and “balloon + weight” conditions ($\left|{{r}_{B+W}-r}_{P}\right|<20\%{\cdot r}_{P}$), while exhibiting a noticeable decrease under the “weight” condition compared to both the “probe-only” condition (${{r}_{P}-r}_{W}>20\%{\cdot r}_{P}$) and the “balloon + weight” condition (${{r}_{B+W}-r}_{W}>20\%{\cdot r}_{B+W}$). The fluorescence rates of these neurons are plotted in Fig. 3e–g, with neuron numbers of N = 28, 22 and 23 in the three experiments, respectively. These neurons were seemingly less active in terms of firing when the mouse was under the weight burden. Among these neurons, neuron #43 (segmentation mask marked in Fig. 3d) consistently showed decreased fluorescence rates under the “weight” condition across all three experiments. Its fluorescence rates are highlighted in red, and its ΔF/F traces of each session are displayed in Fig. 3h. In contrast, there were some neurons that showed no significant difference between the “probe-only” and “balloon + weight” conditions ($\left|{{r}_{B+W}-r}_{P}\right|<20\%{\cdot r}_{P}$) but exhibited a noticeable increase under the “weight” condition (${{r}_{W}-r}_{P}>20\%{\cdot r}_{P}$ and ${{r}_{W}-r}_{B+W}>20\%{\cdot r}_{B+W}$). The number of these neurons is slightly smaller and the fluorescence rates of these neurons across the three experiments are provided in Fig. S5 in Supplementary Materials. These neurons might have fired more actively when the mouse had to overcome the weight burden.

## Discussion

We established a buoyancy levitation system to alleviate head-mounted weight burden for neural imaging in freely-behaving mice. The experimental results demonstrated that the buoyancy-based method can effectively counteract a weight of up to 7 g when using a regular-size (15 L) balloon inflated with a helium mixture. In our case, the 2P fiberscope weighs only approximately 1 g, which does not require balloon lifting. When including additional components for specific imaging control purposes, the total weight might become a significant burden but can be easily counterbalanced by the reported method. For heavier weights, larger balloons can be employed as needed.

It should be noted that larger balloons have greater inertia. In our balloon levitation system, the balloon is positioned inside a guiding cylinder, which restricts its lateral movement to minimize air resistance when the animal moves, allowing only vertical movement of the balloon. Additionally, the nylon cord of the balloon passes through a piece of Teflon tubing to further ensure that only vertical movement is permitted. The tubing is installed approximately 1.2 m above the open arena. Even when the mouse moves from the center to one corner of the arena (around 18 cm), it only causes a 1.34 cm vertical displacement of the balloon and cord with negligible resistance. In our tests, the mice’s average movement speed was generally below 10 cm/s, and we did not observe any sudden tightening or loosening of the balloon cord caused by rapid movement. One solution to the potential inertia issue associated with a large balloon is using a counterbalance system with a weight mounted on a cantilever or a pulley^32,33^. This approach requires preparing a weight equal to the weight of the specific probe being used, while the balloon-based method allows flexible and continuous volume adjustment to counterbalance an arbitrary weight as needed.

Another important concept that needs to be clarified is that while our method effectively compensates for the additional weight using buoyancy, it does not eliminate the added mass, which is an intrinsic property of an object. The added mass to the animal’s head will lead to a reduced acceleration for a given force according to Newton’s second law, thereby potentially affecting the head dynamics. In our experiments, mice were set free in a 10″ × 10″ open arena. Taking mouse #5 as an example, its maximum acceleration was below 1.5 m/s^2^ (see Fig. S4 and note in Supplementary Materials). For a 6 g added mass, an additional force of 9 mN would be required to generate such an acceleration, which is much smaller than the approximately 50 g force that an adult mouse can generate using its forelimbs^34^. Therefore, the inertial effects introduced by the added mass are expected to be relatively minor. However, for animals undergoing higher accelerations or wearing larger head-mounted masses, the inertia introduced by the additional mass might markedly alter their motion behaviors/dynamics. Our current system does not directly measure head dynamics. Future studies could incorporate an accelerometer^35,36^ to obtain precise measurements of linear and angular acceleration, enabling a more quantitative analysis of these effects due to the added mass (and associated inertia).

The performance of the system was evaluated by conducting neuron imaging in freely-behaving mice under three conditions: (1) Probe-only, (2) Balloon + Weight and (3) Weight. Analyses of five mice’s motion behavior revealed that the animals had similar freedom of movement (walking distance, turning angle, linear velocity and angular velocity) when using a balloon to lift the additional head-mounted 6 g weight compared to when only a fiberscope was mounted on their heads. In contrast, the mice moved less and needed more rest without a balloon to alleviate the weight burden.

Additionally, we investigated the impact of head-mounted weight burden on mouse’s motion behavior and neural activities by performing three repeated neuroimaging experiments on a given mouse (#5). Consistently, the mouse moved more actively and was more likely to change its movement state (linear and angular accelerations) when relieved from the weight burden. By analyzing calcium dynamics in identified neurons, we observed that some neurons showed decreased fluorescence rates under the “weight” conditions. These neurons might be less active when the mouse was subjected to weight burden. In contrast, some neurons exhibited increased fluorescence rates under the “weight” conditions, indicating they might be more active when the mouse had to cope with the weight burden. To the best of our knowledge, there is no published report about weight-sensitive neurons. Further investigation can explore the direct effects of weight burden on specific brain regions and enhance our understanding of the connection between head or neck muscles and corresponding neurons.

It is worth noting that our system is not limited to 2P brain imaging in mice. It can be applied to any freely moving animal experiments, involving various probes such as miniscopes, tetrodes, electrode arrays, cameras, or other devices, and can be used for rats or even larger animals. We anticipate that our system will serve as a valuable tool for alleviating the weight burden on the animal’s head, offering new opportunities for studying neural circuit function under naturalistic conditions.

## Electronic supplementary material

Below is the link to the electronic supplementary material.

Supplementary Material 1

## Data Availability

The datasets used and/or analyzed during the current study are available from the corresponding author on reasonable request.
