# Correction: Alleviating head-mounted weight burden for neural imaging in freely-behaving rodents

**DOI:** 10.1038/s41598-025-18189-2

**Published:** 2025-09-09

**Authors:** Yuehan Liu, Jing Zhang, Cheng-Yu Lee, Haolin Zhang, Xingde Li

**Affiliations:** 1 Department of Electrical and Computer Engineering, Johns Hopkins University, Baltimore, MD 21218 USA; 2 Department of Biomedical Engineering, Johns Hopkins University, Baltimore, MD 21205 USA

Correction to: *Scientific Reports* 10.1038/s41598-025-04300-0, published online 31 May 2025

The original version of this Article contained an error in the spelling of the author Cheng-Yu Lee which was incorrectly given as Cheng-Yu Li.

In addition, a grant number was omitted from the Acknowledgements section. Consequently,

“The authors would like to acknowledge the funding support from the National Institutes of Health (NIH) under grant # R01EB033364 (Li). We also thank Aurelius Jing for helping with the motion processing pipeline.”

now reads:

“The authors would like to acknowledge the funding support from the National Institutes of Health (NIH) under grant # R01EB033364 (Li) and # R21EB035306 (Yang). We also thank Aurelius Jing for helping with the motion processing pipeline.”

The original Article has been corrected.

