# Supplementary material for: Alleviating head-mounted weight burden for neural imaging in freely-behaving rodents
Source: Sci Rep. 2025 May 31;15:19175. doi: 10.1038/s41598-025-04300-0 (PMC12126526; doi:10.1038/s41598-025-04300-0)
Supplement: Supplementary file 1 — Supplementary Material 1 [file 41598_2025_4300_MOESM1_ESM.docx]

**Alleviating Head-mounted Weight Burden for** **Neural Imaging in Freely-behaving Rodents Using a Helium-filled Balloon**

**Yuehan Liu,^1^ Jing Zhang,^2^ Cheng-Yu Li,^1^ Haolin Zhang,^2^ and Xingde Li^1,2,*^**

*^1^ Department of Electrical and Computer Engineering, Johns Hopkins University, Baltimore, Maryland 21218, USA*

*^2^ Department of Biomedical Engineering, Johns Hopkins University, Baltimore, Maryland 21205, USA*

*** [*xingde@jhu.edu*](mailto:xingde@jhu.edu)

**Supplementary Figures and Notes**


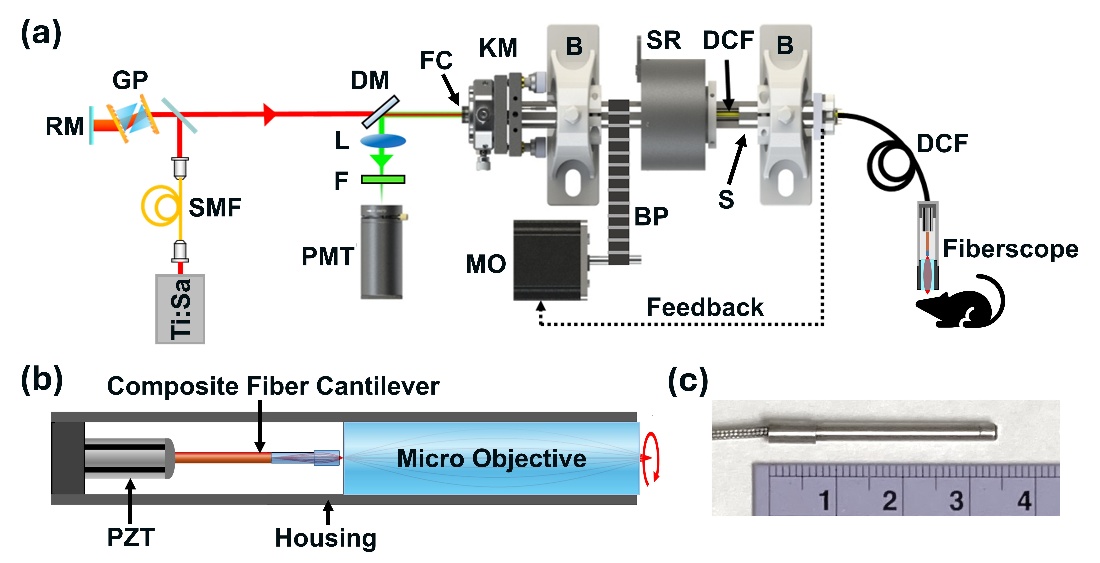


**Figure S1.** The schematics of 2P system and 2P fiberscope. (a) Schematic of optoelectrical commutator along with 2P fiberscope imaging system. SMF: single-mode fiber; RM: roof mirror; GP: GRISM pair for dispersion compensation; DM: dichroic mirror; L: lens; F: optical filter; PMT: photomultiplier tube; FC: fiber collimator; KM: kinematic mount; MO: stepper motor; B: bearing; BP: belt/pulley; SR: slip ring; DCF: double-clad fiber. (b) Schematic of a 2P fiberscope. (c) Photograph of a fiberscope.


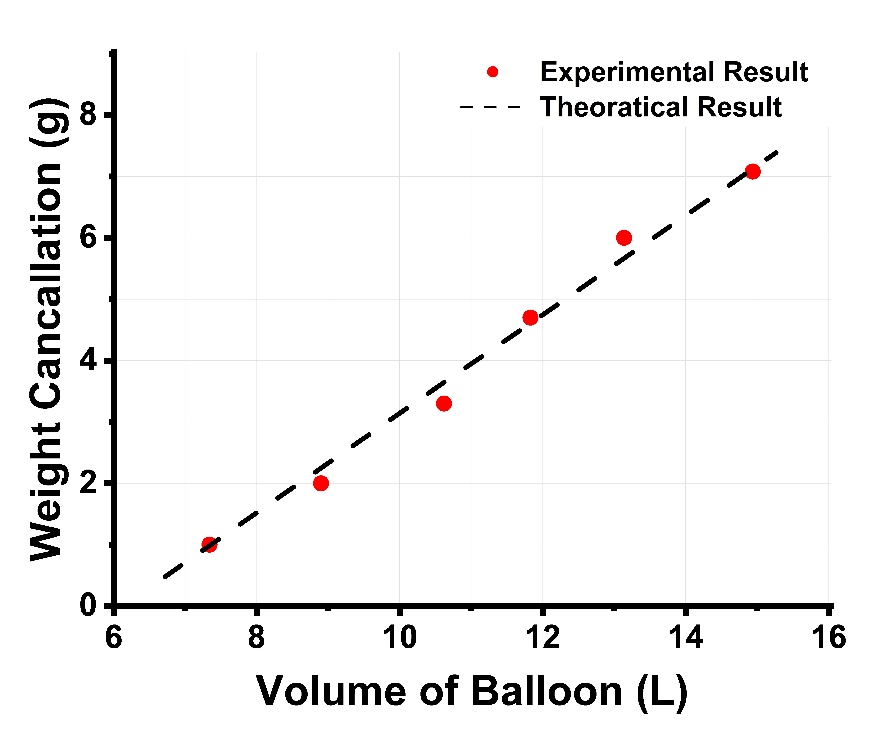


**Figure S2.** The relationship of weight cancelled by the buoyancy verse the volume of the helium balloon. The red dots show the experimental results, and the dashed line illustrates the theoretically calculated values.

**Note:**

**Figure S2** plots the relationship between the weight cancelled by the buoyancy and the volume of the balloon, showing both the theoretically calculated values and the experimental measured results. The measurements were performed by placing weights on a precision electronic scale. The weight cancellation was confirmed only when the scale read 0.0 g, with a helium balloon lifting the weight. The volume of the balloon was determined by measuring its diameter and height. The figure illustrates that the actual measurement results closely match the theoretical predictions, with minor deviations likely due to inaccuracies in measuring the balloon's dimensions.


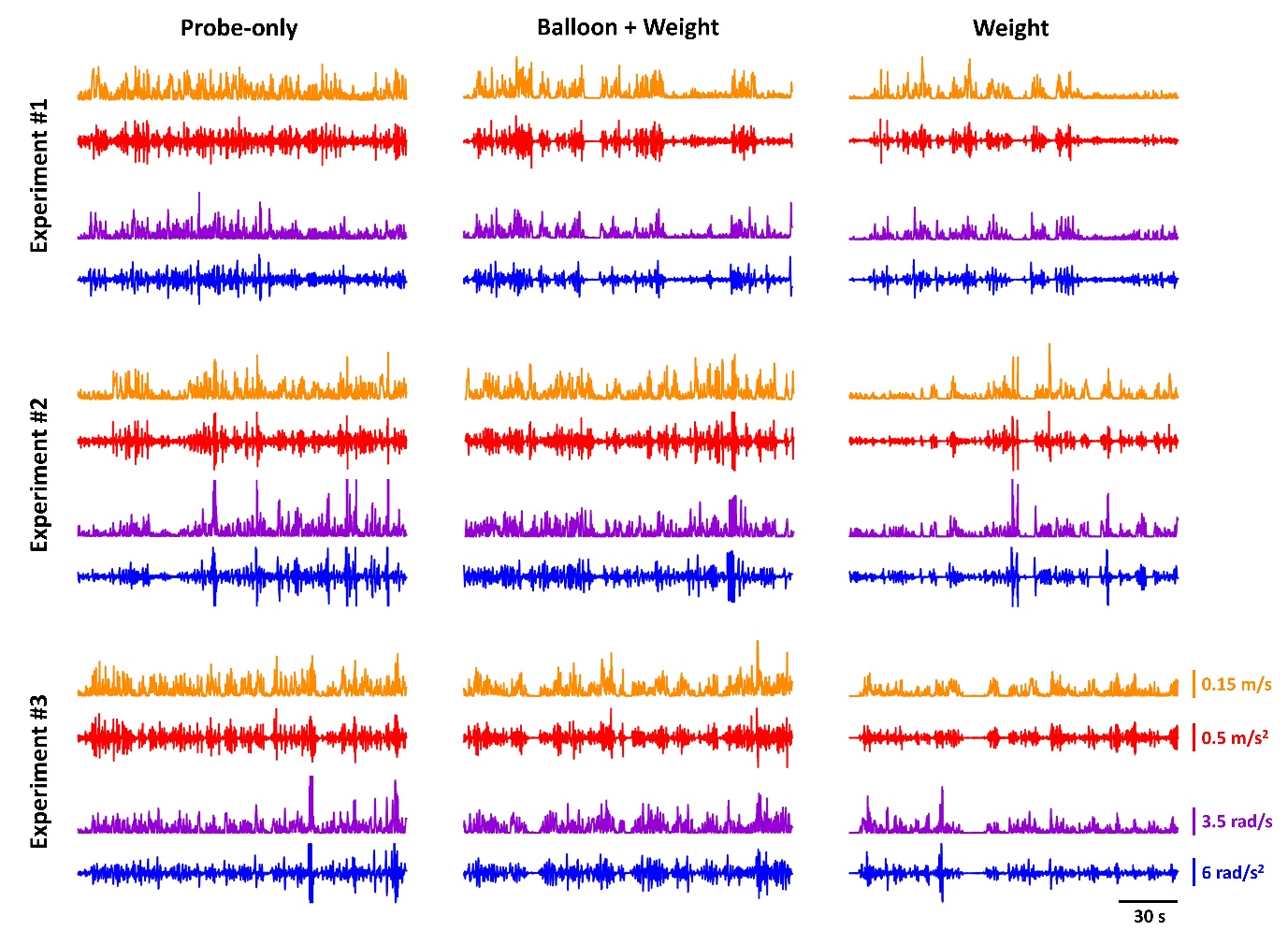


**Figure S3.** Mouse #5’s velocity and acceleration traces within each session under different conditions from three experiments. Orange: linear velocity traces. Red: linear acceleration traces. Purple: angular velocity traces. Blue: angular acceleration traces.


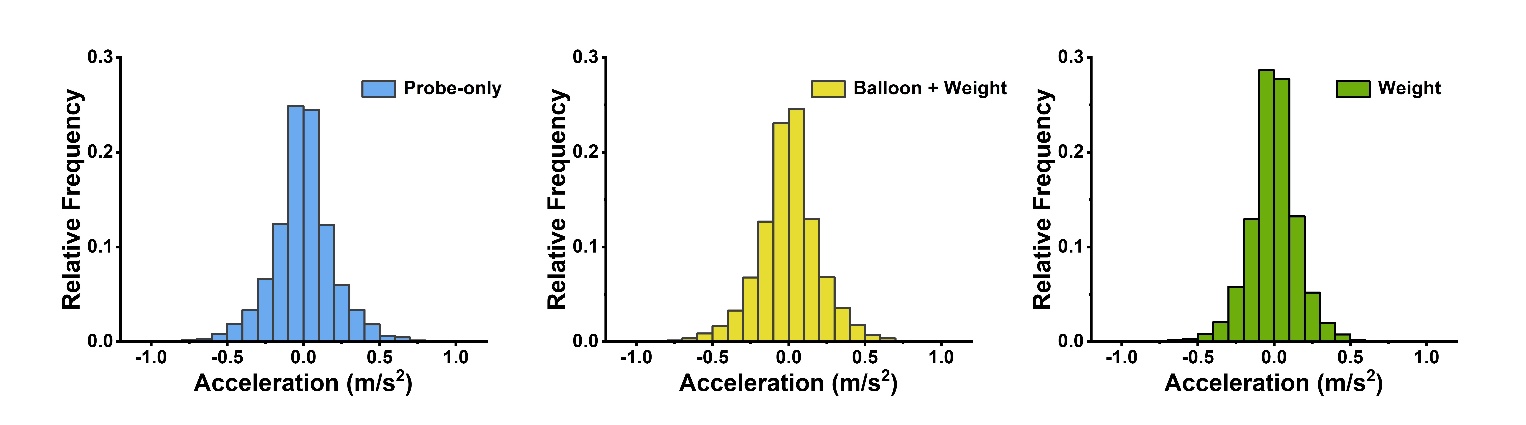


**Figure S4.** Mouse #5’s acceleration distribution histograms during the locomotion periods across three experimental sessions (#1, #2 and #3, as shown in Figure S3) under different conditions (blue: probe-only, yellow: balloon + weight, green: weight).

**Note:**

We distinguished the locomotion and non-walking (grooming or resting) periods of mouse #5 by setting a velocity threshold. Here, we selected 1 cm/s as the threshold, considering both the activity level of the mouse and the size of the arena [1]. The periods identified based on this threshold corresponded well with the mouse’s movement observed in the video footage. We used DeepLabCut [2] to track the mouse’s position in each frame (25 fps) and computed the velocity and acceleration traces for each experimental session. Our calculated accelerations are comparable to those reported in other studies [3-5]. More accurate results could be obtained by direct measurements using an accelerometer [6, 7], which would be incorporated in future studies.


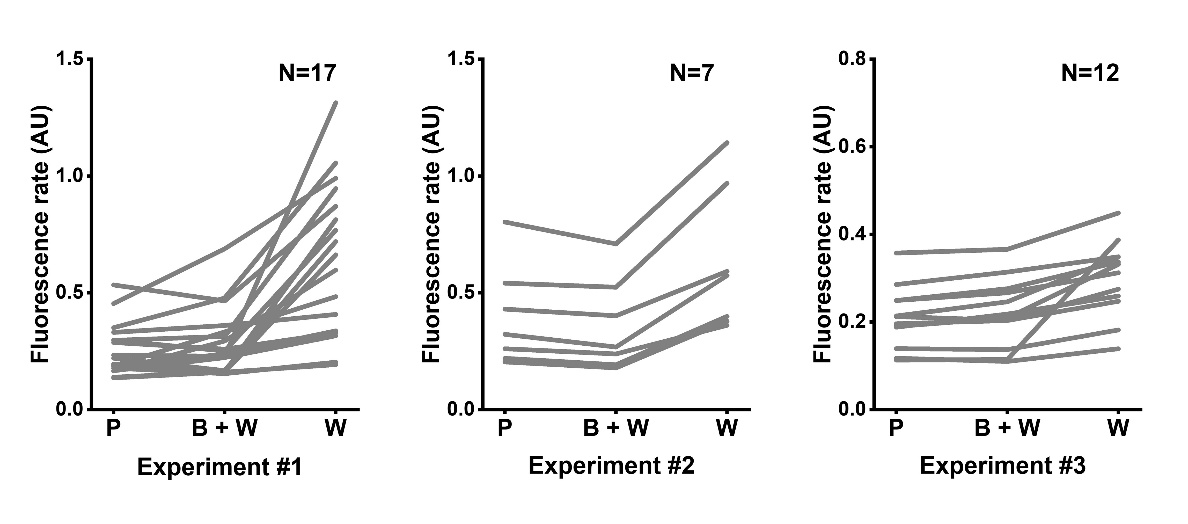


**Figure S5.** Fluorescence rates of neurons under three different conditions (P: probe-only; B + W: balloon + weight; W: weight) in Experiment #1 #2 and #3. These neurons did not show significant differences in their fluorescence rates under the “probe-only” and “balloon + weight” conditions, while showing obvious increase under the “weight” condition. Numbers of these neurons are 17, 7 and 12 in three experiments, respectively.

**References:**

1. T. Del Rosario Hernández, N. R. Joshi, S. V. Gore, J. A. Kreiling, and R. Creton, "An 8-cage imaging system for automated analyses of mouse behavior," Scientific Reports **13**, 8113 (2023).

2. A. Mathis, P. Mamidanna, K. M. Cury, T. Abe, V. N. Murthy, M. W. Mathis, and M. Bethge, "DeepLabCut: markerless pose estimation of user-defined body parts with deep learning," Nature Neuroscience **21**, 1281-1289 (2018).

3. C. de Lombares, E. Heude, G. Alfama, A. Fontaine, R. Hassouna, C. Vernochet, F. de Chaumont, C. Olivo-Marin, E. Ey, and S. Parnaudeau, "Dlx5 and Dlx6 expression in GABAergic neurons controls behavior, metabolism, healthy aging and lifespan," Aging (Albany NY) **11**, 6638 (2019).

4. J. Hope, T. M. Beckerle, P.-H. Cheng, Z. Viavattine, M. Feldkamp, S. M. Fausner, K. Saxena, E. Ko, I. Hryb, and R. E. Carter, "Brain-wide neural recordings in mice navigating physical spaces enabled by robotic neural recording headstages," Nature Methods **21**, 2171-2181 (2024).

5. Y. Sun, J. Zhang, Q. Wang, and J. Ni, "RpiBeh: a multi-purpose open-source solution for real-time tracking and behavior-driven closed-loop interventions in rodent neuroethology," bioRxiv, 2024.2012. 2002.626497 (2024).

6. S. Venkatraman, X. Jin, R. M. Costa, and J. M. Carmena, "Investigating neural correlates of behavior in freely behaving rodents using inertial sensors," Journal of neurophysiology **104**, 569-575 (2010).

7. M. O. Pasquet, M. Tihy, A. Gourgeon, M. N. Pompili, B. P. Godsil, C. Léna, and G. P. Dugué, "Wireless inertial measurement of head kinematics in freely-moving rats," Scientific reports **6**, 35689 (2016).
